# Editorial: Proceedings From ACCM19: Cell Cycle, DNA Damage Response and Telomeres

**DOI:** 10.3389/fcell.2020.00805

**Published:** 2020-09-03

**Authors:** Andrew Burgess, C. Elizabeth Caldon

**Affiliations:** ^1^Cell Division Lab, ANZAC Research Institute, Concord Hospital, Concord, NSW, Australia; ^2^Faculty of Medicine and Health, Concord Clinical School, University of Sydney, Sydney, NSW, Australia; ^3^The Kinghorn Cancer Centre, Garvan Institute of Medical Research, Darlinghurst, NSW, Australia; ^4^Faculty of Medicine, St. Vincent's Clinical School, UNSW Sydney, NSW, Australia

**Keywords:** cell cycle, telomere, DNA damage, mitosis, JNK, radiation, chemotherapy, PP2A

## Introduction

The 19th ACCM meeting was held in Sydney, Australia, from the 17–19th of June 2019. The Australian Cell Cycle Meeting (ACCM) began in the late 1990s as a small Australian based workshop, to bring together cell cycle researchers from across the country once a year. A strong focus was placed on providing students and post-docs with the opportunity to give oral presentations of their work and establish new collaborations. It continued in this fashion for over a decade, establishing itself as a must-attend event for the local cell cycle community. In 2015, we expanded the meeting to incorporate the fields of DNA damage and telomere biology and switched to running every 2 years.

The once small local meeting has now become an internationally and respected meeting that attracts leading researchers from around the world. Our invited research leaders for 2019 were Agata Smogorzewska on genome maintenance (Rockefeller University, New York, USA), Agnel Sfeir on telomere biology (Skirball Institute of Biomolecular Medicine, New York University, Langone Medical Center, USA), Gerry Hanna on radiation oncology (Director of Radiation Oncology Peter MacCallum Institute Victoria, Australia), and Karlene Cimprich on genome stability and DNA replication (School of Medicine, Stanford University, Stanford, CA, USA). Importantly, the meeting maintained its early goal of providing a platform for students and junior scientists to present their research in a friendly and collaborative environment. The next meeting is scheduled for the end of 2021 and will return to Melbourne, where it commenced in 1999.

## Cell Cycle, DNA Repair, and Telomeres: Elements that Underpin Cancer Biology and Treatment

The 2019 ACCM meeting brought together research covering DNA repair, telomere biology, RNA transcription, early developmental biology, cell-polarity and cell-signaling, and big-data. The exploration of these ideas in one forum provided an opportunity for in depth discussion of focused questions with cross-fertilization of ideas from closely aligned research areas.

Two excellent reviews by Sia et al. and Martin and Martin delve into Radiation therapy (RT), which is used to treat more than half of all cancers. With recent technological advancements, RT is responsible for as much as 40% of all cancer cures. The review by Sia et al., examines the types and mechanisms of cell death induced by RT in cancer. RT induces a range of death processes, from classical apoptosis and mitotic cell death through to autophagy, which is mediated by numerous intrinsic pathways in combination with the local microenvironment and radiation specific factors. In their opinion piece, Martin and Martin, contextualize the use of radiation therapy treatment by discussing the notable side effects that many radiotherapy patients face, especially accelerated aging.

This work was complemented by two reviews focused on aspects of lung cancer. Work by Johnson et al., comprehensively examined the role of the oncogene Y-Box protein 1 (YB-1) in driving lung cancer. YB-1 is a multi-function protein capable of regulating transcription, translation, and DNA repair. It is commonly over-expressed in numerous cancers, and it is implicated in driving proliferation, metastasis, and resistance to chemotherapy. In parallel, Gonzalez-Rajal et al. have identified and reviewed the recent breakthrough studies that have shed light on the underlying mechanisms of innate platinum resistance in lung cancer. Notably, platinum chemotherapy is a cornerstone and front-line treatment for many lung cancers including mesothelioma. It is essentially curative in testicular cancer but is hampered by extensive innate resistance in lung cancer, with only 30% of patients responding. In this opinion article, Gonzalez-Rajal et al. highlight how TGFβ signaling, the cell cycle and DNA repair are key central players in regulating platinum resistance in lung cancer.

Three research manuscripts provided key findings on DNA repair and telomeres with respect to cancer biology mechanisms, improved cancer biomarkers, and methodological advancement. One essential pathway that regulates platinum resistance is the Fanconi Anaemia (FA) pathway, which is responsible for removing platinum induced interstrand crosslinks (ICLs) from DNA. Exciting new research by Tan et al. examines how two crucial components of the FA pathway, FANCD2 and FANCI, are regulated by direct phosphorylation by ATR kinase. This protects the FA complex from degradation, thereby ensuring cross-linked DNA is properly repaired. Notably, cancer cells often have defective DNA repair pathways making them susceptible to novel chemotherapies. One such treatment is the RNA polymerase I (PolI) transcription inhibitor CX-5461, which causes DNA damage and is currently in phase I clinical trials for solid tumors. Work from Son et al. demonstrates the number of active rDNA repeats positively correlates with sensitivity to CX-5461 in ovarian cancer cells, and hence may be a potential clinical biomarker for this exciting new chemotherapy. Notably, telomeres protect the chromosome ends from being recognized as DNA double-strand during normal replication. Disruption of telomeres is a hallmark of cancer, and hence the ability to accurately monitor telomere length is a critical assay not only for basic research but also clinical diagnosis. In exciting new research, Kahl et al. demonstrate the Telomere length Combing Assay (TCA), which can accurately measure telomere length in cell populations by pulling DNA fibers out onto glass coverslips using a constant stretching factor.

Telomeres and DNA repair also play essential roles in early developmental biology. Kafer and Cesare provide a comprehensive review of the replication stress and HR repair factors that are essential for early mammalian embryo development covering over 347 genes. Understanding embryo development and the use of developmental systems such as *Drosophila* (Fruit flies) has been essential for discovering and new drivers of cancer. Pre-eminent among these is the c-Jun N-terminal Kinase (JNK) signaling pathway, which plays a multitude of roles from regulating cell proliferation through to survival. Here, La Marca and Richardson provide a compelling review of how the JNK pathway acts as both promoter and inhibitor of tumorigenesis in *Drosophila*. Traditionally, early developmental biology, DNA repair, and cell cycle pathways have focused on the role of kinases, however, the essential role of counterbalancing phosphatases has recently gained prominence. This is highlighted in new research by Panicker et al., where they analyse the role of the major phosphatase PP2A. PP2A is a multi-complex phosphatase, with specificity for substrates controlled by the regulatory subunit (Rogers et al., 2016). Using CRISPR/Cas9, Panicker et al., demonstrate that knocking out the B55α regulatory subunit caused embryonic lethality in mice, due to failed epidermal stratification, highlighting the importance of phosphatases.

## Summary

The ACCM conference and resulting special issue in Frontiers in Cell and Developmental Biology has highlighted the biological complexity that underpins cancer biology and treatment, and that major aspects of this biology are the cell cycle, DNA repair pathways, and telomere biology. A key aspect of our meeting is to bring together what are sometimes diverse fields and encourage the sharing of ideas that can accelerate knowledge gain and implementation. The studies published in this special issue cross over these fields and highlight the impact of cross-fertilization; examples include the importance of developmental biology on understanding of DNA damage pathways, and of how the basic understanding of the detection and repair of DNA lesions could alter cancer therapy.

## Author Contributions

All authors listed have made a substantial, direct and intellectual contribution to the work, and approved it for publication.

## Conflict of Interest

The authors declare that the research was conducted in the absence of any commercial or financial relationships that could be construed as a potential conflict of interest.
